# Prevalence of chronic obstructive pulmonary disease and associated factors among small-holder fish vendors along coastal areas in Tanzania

**DOI:** 10.1186/s12890-023-02576-4

**Published:** 2023-08-02

**Authors:** Brigitha M. Onesmo, Simon H. Mamuya, Mwema Felix Mwema, Jerry Hella

**Affiliations:** 1 Ifakara Health Institute, P. O. Box 78373, Dar es Salaam, Tanzania; 2 School of Life Sciences and Bio-Engineering, The Nelson Mandela African Institution of Science and Technology, P. O. Box 447, Arusha, Tanzania; 3 Mpwapwa Institute of Health and Allied Sciences, The Ministry of Health, P. O. Box 743, Dodoma, Tanzania; 4 Department of Environmental and Occupational Health, School of Public Health and Social Sciences, Muhimbili University of Health and Allied Sciences, P. O. Box 65001, Dar es Salaam, Tanzania; 5 School of Materials, Energy, Water and Environmental Sciences, The Nelson Mandela African Institution of Science and Technology, P. O. Box 447, Arusha, Tanzania

**Keywords:** Chronic obstructive Pulmonary Disease, Fish vendors, Tanzania

## Abstract

**Background:**

In Tanzania little is known about how the respiratory system of small-holder fish vendors is affected by occupational exposure to biomass smoke and other associated factors. This study assessed the prevalence of lung obstruction and associated factors among small-holder fish vendors along coastal areas in Tanzania.

**Methods:**

A cross-sectional descriptive study was conducted in Bagamoyo and Kunduchi fish markets along coastal areas of Tanzania. Environmental air pollutant levels and composition were measured using a hand-held device. A standardized questionnaire was used to assess respiratory symptoms while EasyOne spirometer was used to test for lung function among small-holder fish vendors. Chronic Obstructive Pulmonary Disease (COPD) was defined as FEV1/FVC below the lower limit of normal. Data were analyzed using STATA Version 17. Descriptive statistics was performed and logistic regression analysis was used to determine factors that are associated with poor lung function presented as crude and adjusted odds ratio and their 95% confidence intervals.

**Results:**

A total of 103 participants were included in the study who were predominantly males 82 (79.6%). The participants’ mean age was 35.47 (± 8.77 SD) years. The hourly average concentration levels of PM_1_, PM_2.5_, PM_10,_ and CO exposure during fish frying were 653.6 (± 206.3 SD) μg/m^3^, 748.48 (± 200.6 SD) μg/m^3^, 798.66 (± 181.71 SD) μg/m^3^ and 62.6 (± 12.3 SD) ppm respectively which are higher than the WHO recommended limits. The prevalence of COPD was found to be 32.04% (95% CI 0.23–0.42). Most of the participants reported respiratory symptoms like coughing, wheezing, sputum production and breathlessness during performing their daily activities.

**Conclusion:**

Findings suggest that three out of ten participants had COPD and the major environmental air pollutants (PMs and CO) concentration levels were too high, suggesting that occupational exposure to biomass smoke may be a risk factor. This calls for effective approaches to reduce exposure and prevent known acute and chronic respiratory diseases that are associated with such exposure to air pollutants. Also the study calls for follow up or cohort studies to be conducted in this area.

## Background

Non-communicable diseases (NCDs) have recently become a global public health concern and are estimated to contribute to more than 70% of deaths worldwide [1] which is equivalent to 41 million people who die each year [2]. In low and middle income countries, these NCDs accounts for about 75% of all causes of death and about 86% causes of premature mortality in these countries [2]. The World Health Organization (WHO) has categorized key groups of NCDs including chronic respiratory diseases among others.

Environmental risks, particularly air pollution increases a considerable burden on NCDs [3, 4]. WHO estimated that indoor air pollution resulting from biomass fuel use causes about 4 million premature mortality each year [5]. NCDs are influenced by the people’s lifestyle factors such as unhealthy diets, physical inactivity, tobacco use, the harmful use of alcohol [2], and poor working environments [6]. Air pollution alone puts 90% of people at increased risk for NCDs including cancer, chronic obstructive pulmonary diseases (COPD) and cardiovascular diseases [7]. Studies conducted in 2018 in India reported an increased threat of developing COPD because of air pollutants from use of biomass solid fuels [8]. Air pollutants like PM, NO_2,_ O_3_ and SO_2_ emitted from burning of biomass fuel have the ability to affect the respiratory system and cause respiratory symptoms like phlegm, cough and bronchial hyper responsiveness [9].

COPD imposes a substantial economic burden on providers of health care service and on patients themselves while negatively affecting the daily lives of patients, impairing the wellbeing of an individual, reducing productivity and functional status with a major economic impact [10]. Inefficient combustion of biomass fuel emits high level of air pollutants like PM and CO [11]. Nowadays, the focus of air pollution has risen because particulate matters (PM_2.5_) have the ability to penetrate lung tissue and induce local and systemic effects. Particles with 10 microns diameters or below (PM_10_) and those with 2.5 microns diameter or below (PM_2.5_) may enter deeply in the lungs, hence causing damage to health [12].

Evidence has shown that exposure to biomass smokes increase both respiratory and non-respiratory diseases [13]. A longitudinal study conducted in Kenya has established an association between inhalation of polluted air caused by biomass use and acute respiratory infections [14]. For a human to be exposed to pollutants from air depends on indoor and outdoor amounts of air pollutants, the environment and time of exposure in such conditions [15]. Air pollution from cooking places with poor ventilation tends to result in due decline of lung function [8].

Tanzania is committed to achieve the sustainable development goal number 7, by which the country through the ministry of energy and minerals promote use of clean and affordable energy in many ways, including the establishment of Rural Energy Agency (REA) which ensures affordability and availability of electricity in rural areas [16]. Furthermore, the Tanzanian government through health sector strategic plan (2021–2026) they have a plan in strengthening Public Private Partnership (PPP) in addressing the health effects of air pollution from business and commercial activities [17]. However, biomass fuel is still the source of energy in homes and businesses [18] due to its affordability and availability. Few cross-sectional studies have measured the lung function of people at risk in Tanzania who use biomass fuel [19]. A cross-sectional study conducted in Simiyu region in Tanzania, reported 99.5% of the households used biomass fuels for cooking and the prevalence of COPD was estimated at 17.5% [19]. Another study conducted in an informal urban environment reported the prevalence of COPD at 8.13% [20]. A study done in Bagamoyo showed the prevalence of acute respiratory illness were 54% among women involved in cooking [21]. It is hypothesized that fish vendors exposed to biomass smoke, fumes and heat when frying fish are at high risk of respiratory diseases.

In Tanzania there is a legal framework which ensures health and safety in the community and workplaces. The national policy on energy use of 2003 emphasizes on availability of reliable, affordable and environmentally sound energy sources. The policy aims to lower the impact of using biomass fuel (firewood) both in domestic and at workplaces by replacing it with the use of clean energy [22]. Also, the national environmental management Act of 2004 [23] provides the mandate to public health inspectors in both local and central government authorities to enforce and monitor air quality standards in all premises. Fish processing settings are among the premises that are required to adhere with the stipulated standards as provided in the national Air quality standard regulation of 2007 [24]. Despite the presence of legal framework, however its implementation is challenging due to inadequate coordination, and facilities to monitor air quality in workplaces. Thus, there is a need to rethink on the best way that can ensure effective monitoring of air quality in workplaces.

There is limited information in the country on the occupational impact from air pollution exposure among small-holder fish vendor’s who almost always rely on biomass fuel for frying fish prior to selling them. This study assessed the prevalence of COPD and associated factors among small-holder fish vendors along coastal areas in Tanzania. The study would provide information about the lung health of participants which is valuable information for decision makers, medical practitioners and health-care planners to promote quality of life, better health and reduce or prevent morbidity and mortality from COPD.

## Methods

### Study design, study area and study population

The study was a cross-sectional descriptive study and was conducted in Bagamoyo and Kunduchi fish markets along the coast of the Indian Ocean in Tanzania. Bagamoyo fish market is located in Dunda ward in Bagamoyo District on the coast of the Indian Ocean, approximately 75 km North of Dar es Salaam. The district lies between 6° 26′ South of the equator and 38° 54′ East of prime meridian. Kunduchi fish market is located in Kunduchi ward in Kinondoni District in Dar es Salaam. The local people engage mainly with fishing activities at the Indian Ocean, and sell them at these markets which are the center for commercial activities. Fish are being sold either raw or fried. Those who engage with frying fish for selling purposes use firewood as the main source of energy. They work in an area with poor ventilation thus the exposure to smoke becomes very high.

### Sampling and sample size consideration

Given a previous study on an informal urban environment [20] which reported the prevalence of COPD at 8.13% the sample size was estimated at 114 participants. This was because it was in an informal urban areas same to the current study area. Sample size was then estimated using a power command on STATA where we anticipated the prevalence of COPD to double because the exposure at the study area were much higher than the one reported by the previous study. Hence the sample size was estimated at 109 participants with an addition of 5% non-respondent which yielded 114 participants obtained by the systematic sampling for which all fish vendors who were 18 years and above were listed and every third member were selected for the study. We therefore reached 103 participants as others refuse to participate in the study whereby, 37 were from Kunduchi fish market and 66 from Bagamoyo fish market. All 103 participants performed the spirometry test. All fish vendors were Africans from different tribes in Tanzania.

Participants were provided with a written informed consent to seek for voluntary participation in the study in which they were assured confidentiality and anonymity and informed to be free to withdraw anytime they feel. Description of the objectives of the study, potential benefits and risks were given to the study participants.

Unique numbers were used to identify participants’ data and no individual names or other identifying data were uploaded in the database. All data collected were accessible to researchers only and not accessible to unauthorized personnel.

### Air pollution monitoring

At both markets, the composition and levels of air pollutants were monitored, particularly fine particles, carbon monoxide, sulfur dioxide at the workplace. The measurement of particulate matter (PM) emission levels at Bagamoyo and Kunduchi fish markets was conducted using a hand-held Intelligent Air Detector (Igeress series). During measurements, the air detector was placed approximately 1-meter-high to capture the inhalable air within the breathing zone of fish vendors but was also placed within 1 m of the combustion zone to avoid it being too close to fire as it can be damaged. The amount of air pollutants in the working environment was measured on a real time basis by repeated measurements every five minutes for 1 h. Samples were taken by determining the spatial configuration of the market to capture at least 95% of the area [19] by sample on sides of market and at the center three times in a day i.e., before the fish vendors start their activities in the morning, during fish frying process and after their work to obtain information concerning the variation of the particles with time. Prior to data collection every day the calibration of equipment was conducted. The amount of these pollutants was then compared with the WHO limits and guidelines [25].

### Respiratory symptoms among study population

A modified St. George respiratory questionnaire (SGRQ) [26] was used to assess respiratory symptoms including shortness of breath, phlegm, wheezing, coughing, nasal congestion. The questionnaire also assessed disturbances to daily physical activities due to the respiratory problems, and the impact of it to psychosocial function. Respiratory symptoms were measured as symptoms of either coughing, phlegm production, shortness of breath, wheezing, or activities which make the participant feel breathlessness like walking uphill, walking up a flight of stairs, playing sports or other physical activity over the past three months. Participants may experience respiratory symptoms as either many days in a week, few days in a week, few days in a month, or only when having respiratory infection or not experiencing respiratory symptoms at all. Those who reported to have respiratory symptoms for 3 or more days in a week were considered with higher respiratory symptoms. Also if participants experience any of the mentioned respiratory symptom in the past three months for 3 or more than three days consecutives, the person was considered with chronic respiratory problems. Other confounding factors like age, duration of work, Body Mass Index, smoking history, history of asthma, and tuberculosis were assessed and adjusted in the analysis. The questionnaire was administered face to face with the fish vendors. The questions were translated from English version to Swahili version and back using different research assistants and then administered using Swahili language.

### Lung function test among study population

All participants were tested for pulmonary functions using a calibrated EasyOne diagnostic Spirometer (ndd Medizintechnik AG 8005 Zurich, Switzerland) after explaining the process involved to each participant. Age was recorded in full years and physical measurements were conducted by measuring height with a tape measure and body weight with ZOVEC digital weighing scale. The spirometry test was conducted according to European Respiratory Society (ERS) and American Thoracic Society (ATS) standards [27]. Additionally, participants were instructed not to smoke cigarettes for at least one hour before the spirometry test, not to wear tight clothes and not to take any large meal two hours before doing the test. The test was carried out after the participant was in a sitting position, then the participant was instructed to take a deep breath followed by forced rapid exhalation into a disposable mouthpiece that is connected to a spirometer. Each participant was instructed to blow three acceptable maneuvers in a spirometer and the highest values for forced vital capacity (FVC) and for forced expiratory volume (FEV1) was considered the best and used in analysis. Participants were allowed to rest 2 min between each blow. The FEV1 and FVC was used to calculate the Tiffeneau index as the ratio of FEV1/FVC. The spirometry values higher than the lower limit of normal were considered normal [28] while values below the lower limit of normal were considered abnormal. This was used to characterize participants as having obstructive lung disease (i.e., COPD), restrictive lung disease, mixed pattern or normal. For those who had FEV1/FVC > 70% were considered normal and those who had FEV1/FVC < 70% were considered with obstructive lung diseases, while those who had FVC < 80% were considered with restrictive lung diseases. Participants with FEV1 > 70% were considered with mild lung obstruction, while those with FEV1 between 60 and 69% were considered with moderate lung obstruction, and those with FEV1 < 60% were considered with severe lung obstruction.

COVID-19 preventive measures were followed by using a disposable mouth piece to each participant during the spirometry test, and also the test was conducted to one participant at a time while the research team was wearing a mask. Also hand sanitizers were available during the research activity. The study sites had enough space to ensure social distancing of study participants during spirometry testing.

### Data management and analysis

Data was captured using an electronic platform OpenDataKit (ODK) and was managed in real-time using the same platform. Data was stored in a state-of-the-art database with established backup protocols at Ifakara Health Institute. Data was then exported as .csv files and analyzed using Stata Corps software version 17. All data were imported into Stata for analysis. Descriptive statistics was performed and presented as frequency and proportion as appropriate. Additionally, measures of central tendency were used to summarize the information as mean with standard deviation. Logistic regression analysis (univariate and multivariate models) was used to determine factors that are associated with COPD and presented as crude and adjusted odds ratio and their 95% confidence intervals. A stepwise backward selection of variable was used to arrive to a final logistic regression model with Akaike Information Criterion being used to compare between models.

## Results

### Study population characteristics

The demographic characteristics of 103 participants captured sex, age, education level, duration of work, working hours per day, working days per week, Body Mass Index and smoking history are presented in Table 1.

Out of 103 participants 37 (35.92%) and 66 (64.08%) participants were from Kunduchi and Bagamoyo fish markets respectively. Majority of participants were males 82 (79.6%). The average age of participants was 35.47 (± 8.77 SD) years. About 45 (43.7%) of the participants were between 30 and 39 years of age. Most of the participants 77 (74.8%) had primary level of education, while 17 (16.5%) had ordinary level of education and 9 (8.7%) were with no formal education.

At both markets, all fish vendors (100%) practice open burning/traditional open fires using firewood in frying fish for selling purposes. The long-term exposure to firewood smoke is high, as about 38 (36.9%) of participants have used firewood for frying fish for about five to ten years while 26 (25.2%) of the participants have been exposed to firewood smoke for more than ten years. About 43 (41.8%) and 33 (32%) of the participants reported to work seven days and six days per week respectively.

The current health status of participants was 90 (87.4%) fair, 11(10.7%) good and 2(1.9%) poor. The mean BMI for all participants was 24.18 (± 5.02 SD) kg/m^2^. Most women had higher BMI with a mean of 28.5 (± 6.9 SD) kg/m^2^ and male had a mean BMI of 23.07 (± 3.7 SD) kg/m^2^. All females who participated in this study had no history of smoking. Current smokers were 22 (21.4%), while ex-smokers were 11(10.7%).

**Table 1:** Demographic characteristics of the study population

Characteristics	Total n = 103	Male n = 82 (79.6%)	Female n = 21 (20.4%)	P-value
**Age in years**				0.05
19–29	30 (29.1%)	28 (34.2%)	2 (9.5%)	
30–39	45 (43.7%)	35 (41.7%)	10 (47.6%)	
40–49	19 (18.5%)	11 (13.4%)	8 (38.1%)	
50–59	7 (6.8%)	6 (7.3%)	1 (4.8%)	
60–69	2 (1.9%)	2 (2.4%)	0 (0%)	
**Education level**				0.25
Informal education	9 (8.7%)	9 (10.9%)	0 (0%)	
Primary level	77 (74.8%)	59 (71.9%)	18 (85.7%)	
Ordinary level	17 (16.5%)	14 (17.1%)	3 (14.3%)	
**Duration of work**				0.74
Less than a year	5 (4.9%)	4 (4.9%)	1(4.8%)	
One to five years	34 (33%)	25 (30.5%)	9 (42.9%)	
Five to ten years	38 (36.9%)	31 (37.8%)	7 (33.3%)	
More than ten years	26 (25.2%)	22 (26.8%)	4 (19.1%)	
**Working hours per day**				0.22
Less than five hours	3 (2.9%)	3 (3.7%)	0 (0%)	
Five to eight hours	57 (55.3%)	48 (58.5%)	9 (42.9%)	
More than eight hours	43 (41.8%)	31 (37.8%)	12 (57.1%)	
**Working days per week**				0.002
Less than three days	1 (0.9%)	0 (0%)	1 (4.8%)	
Four days	12 (11.7%)	4 (4.9%)	8 (38.1%)	
Five days	14 (13.6%)	14 (17.1%)	0 (0)	
Six days	33 (32%)	25 (30.5%)	8 (38.1%)	
Seven days	43 (41.8%)	39 (47.6%)	4 (19.1%)	
**BMI (kg/m**^**2**^)				0.003
Underweight	3 (2.9%)	3 (3.7%)	0 (0%)	
Normal	64 (62.1%)	57(69.5%)	7 (33.3%)	
Overweight	26 (25.2%)	19 (23.2%)	7 (33.3%)	
Obese	10 (9.7%)	3 (3.7%)	7 (33.3%)	
**Previous TB history**	3 (2.91%)	3 (2.91%)	0 (0%)	0.37
**Smoking History**				
Never smoked	70 (68.0%)	49 (61.3%)	21 (100%)	0.04
Current smokers	22 (21.4%)	22 (26.8%)	0 (0%)	0.01
Ex-smokers	11(10.7%)	11 (13.4%)	0 (0%)	0.04
Packs per year	0.76 (SD 9.88)	0.97 (SD 4.33)	0 (0%)	0.91

Data are presented as n (%) and mean(SD), unless otherwise stated.

BMI: Body Mass Index.

Comparison by chi-square test.

### Environmental air pollution monitoring

The two fish markets (Bagamoyo and Kunduchi fish markets) were monitored for air pollutant concentrations. Main air pollutants in the two markets were Particulate matter (PM_1_, PM_2.5_ and PM_10_) and Carbon monoxide (CO). The average hourly concentrations of main air pollutants are presented in Table 2. The concentration of air pollutants levels before and after fish frying were very low, while the concentration of air pollutant levels during fish frying where too high especially for PMs and CO. This study was not powered enough to examine if biomass exposure pollutants is a risk factor for having chronic lung obstruction. However, these pollutant concentrations provide useful descriptions of the study area.

**Table 2:** Mean pollutant concentration levels before and during fish frying

Pollutants	Before	95% CI	During	95% CI	P-value
PM_1_ (μg/m3 )	14.8 (SD 3.8)	(12.6–16.9)	653.6 (SD 206.3)	(534.5-772.7)	< 0.001
PM_2.5_ (μg/m3 )	19.1 (SD 5.1)	(16.1–22.0)	748.5 (SD 200.6)	(632.7-864.3)	< 0.001
PM_10_ (μg/m3 )	22.7 (SD 5.8)	(19.4–26.1)	798.7 (SD 181.7)	(693.7-903.6)	< 0.001
C0 (ppm)	0 (SD 0)	(0–0)	62.6 (SD 12.3)	(55.5–69.7)	< 0.001
S0_2_ (ppm)	0.001 (SD 0.00)	(-0.001-0.003)	0.08 (SD 0.09)	(0.03–0.13)	0.004
O_3_ (ppm)	0.02(SD 0.03)	(0.003–0.042)	0.05 (SD 0.04)	(0.02–0.08)	< 0.001
Temperature	27.4 (SD 2.1)	(26.2–28.5)	31.8 (SD 1.8)	(30.7–32.8)	< 0.001
Humidity	65.5 (SD 5.2)	(62.5–68.4)	52.4 (SD 4.8)	(49.6–55.2)	< 0.001

Data are presented as mean (SD).

PM: Particulate Matter; CO: Carbon monoxide; SO_2_: Sulfur dioxide; O_3_: Ozone.

Comparison by t-test.

### Respiratory symptoms of small-holder fish vendors

**Fig. 1:**
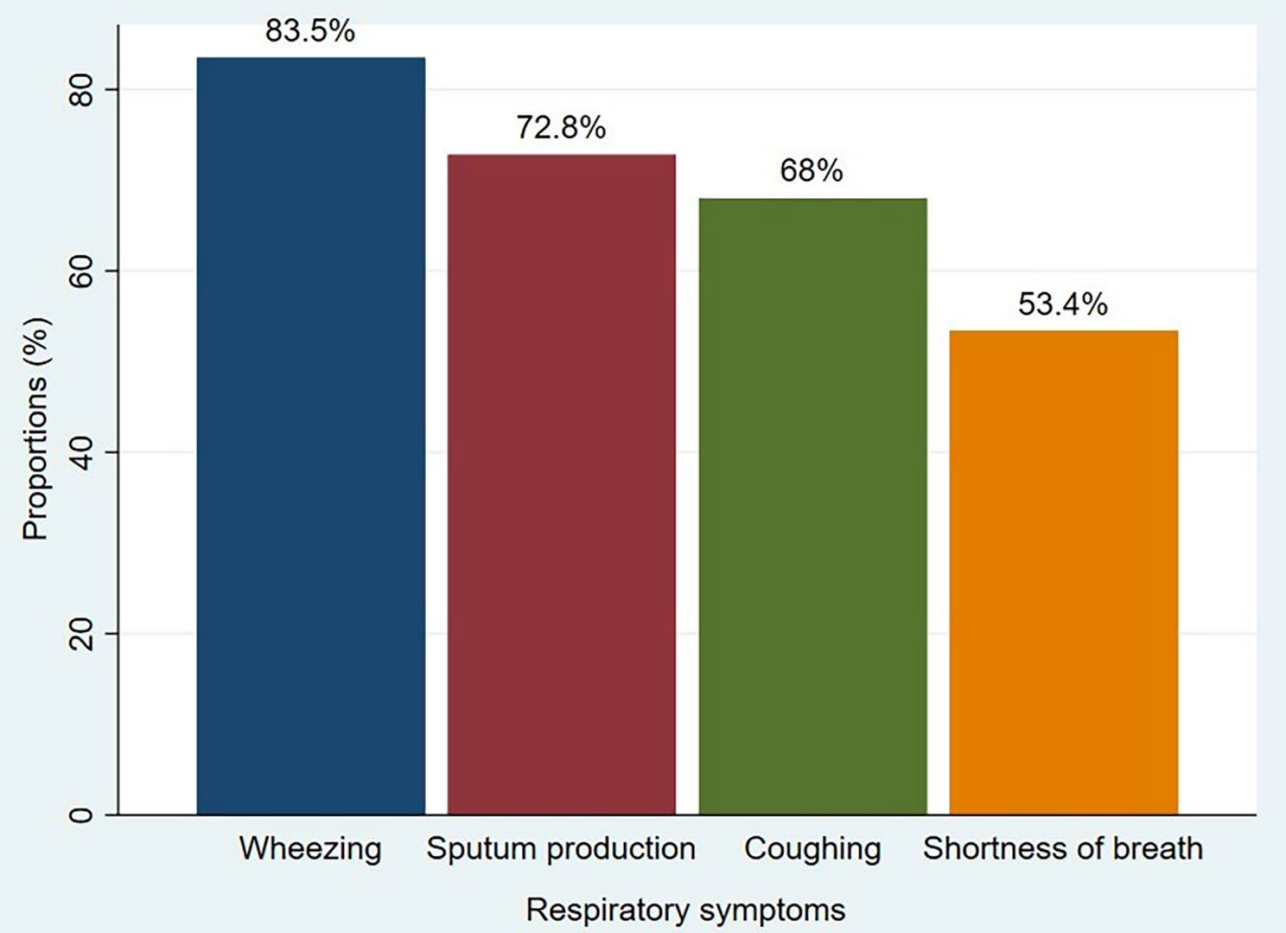
Some common reported respiratory symptoms

The study enquiry participants on the occurrence of respiratory symptoms. Most of the participants reported that they had experienced respiratory symptoms over the past three months. Coughing was reported as the main respiratory symptom by 70 (68%) of participants and sputum production was reported by 75 (72.8%) while wheezing was reported by 86 (83.5%) and shortness of breath was reported by 55 (53.4%) (Fig. 1). About 32 (31.07%) reported that they walk slow than others of the same age when on level ground, 52 (50.49%) participants reported that they had to stop for breathing when walking on hurry and 75 (72.8%) reported breathlessness when walking flight of stairs, while 79 (76.7%) reported breathlessness when walking uphill and 15 (14.6%) reported breathlessness when walking on level ground.

### Lung function fitness of small-holder fish vendors

Using FEV1/FVC lower limit of normal (LLN) [27]about 32.04% (95% CI 0.23 to 0.42) had COPD. About 81 (78.64%) were categorized as having mild COPD, 11 (10.7%) moderate COPD and 11 (10.7%) had severe COPD (Table 3).

Of the 103 participants who performed spirometry, about 19 (18.45%) were suspected of having restrictive lung disease, 19 (18.45%) were suspected as having mixed lung disease (both obstructive and restrictive lung disease) and 50 (48.54%) were normal.

**Table 3:** Lung function values of study participants

Lung function values	Total Mean ± SD	Male Mean ± SD	Female Mean ± SD	P-value
FEV1 (L)	2.5 ± 0.8	2.7 ± 0.6	1.7 ± 0.7	0.06
FEV1% predicted	83.3 ± 21.2	85.8 ± 17.2	71.9 ± 27.8	0.04
FVC (L)	3.5 ± 1.2	3.8 ± 1.1	2.5 ± 1.0	0.24
FVC % predicted	97.5 ± 27.9	99.1 ± 25.9	91.5 ± 34.7	0.09
FEV1/FVC	73.3 ± 14.6	74.5 ± 13.5	68.4 ± 17.8	0.40
**Lung obstruction category**	**Total** **N (%)**	**Male** **N (%)**	**Female** **N (%)**	0.001
Mild	81 (78.6%)	69 (84.1%)	12 (57.1%)	
Moderate	11 (10.7%)	9 (10.9%)	2 (9.5%)	
Severe	11 (10.7%)	4 (4.9%)	7 (33.3%)	

Data are presented as mean + SD and n (%)

Comparison by chi-square test.

In multivariate logistic regression analyses, males had reduced odds for COPD by 75% (AOR 0.25, 95% CI 0.06–1.26) as compared to females, although this observation were non-significant (Table 4). Interestingly, those who were underweight have increased odds for COPD while those who were obese have reduced odds of COPD (AOR 0.09, 95% CI 0.01–0.91) (Table 4). Previous history of TB treatment was associated with a 75% increase in the odds for developing COPD (AOR 1.75, 95% CI 0.2-15.38), again this finding was not statistically significant. Intuitively, increased number of days one worked in frying fish was associated with incremental higher odds for COPD (Table 4). Lastly, participants who reported feeling out of breaths while walking on level ground or when climbing stairs had increased odds for developing COPD with AOR 3.08 and 1.2 respectively (Table 4).

**Table 4:** Univariate and multivariate analysis for predictors of COPD

Characteristics	No COPD	COPD	Univariate model			Multivariate model		
			Unadjusted OR	95% CI	P value	Adjusted OR	95% CI	P value
Age, years	35.2 (8.8)	36.1 (8.8)^**^	1.01	0.96–1.06	0.62	-		
**Gender**								
Female	13 (18.57)	8 (24.24)	1	-	-	1	-	-
Males	57 (81.43)	25 (75.76)	0.71	0.26–1.93	0.51	0.25	0.06–1.16	0.076
**BMI category**								
Normal	43 (61.43)	21 (63.64)	1	-	-	1	-	-
Underweight	1 (1.43)	2 (6.06)	4.10	0.35–47.77	0.261	6.91	0.61–78.53	0.119
Overweight	17 (24.29)	9 (27.27)	1.08	0.41–2.84	0.869	0.90	0.33–2.46	0.838
Obese	9 (12.86)	1 (3.03)	0.23	0.03–1.92	0.173	0.09	0.01–0.91	0.04
**Education level**								
No/informal education	7 (10)	2 (6.06)	1	-	-	-		
Primary level	54 (77.14)	23 (69.70)	3.11	0.5-19.54	0.226	-		
Secondary level	9 (12.86)	8 (24.24)	1.49	0.29–7.73	0.634	-		
**Smoking cigarettes**								
No	48 (68.57)	22 (66.67)	1	-	-	1	-	-
Yes	22 (31.43)	11 (33.33)	1.09	0.45–2.64	0.847	0.96	0.31–2.95	0.94
**History of TB treatment**								
**No**	68 (97.1)	32 (96.9)	1	-	-	1	-	-
Yes	2 (2.86)	1 (3.03)	1.06	0.09–12.15	0.961	1.75	0.2-15.38	0.62
**Exposure to fish frying**								
Less than 1 year	2 (2.86)	3 (9.09)	1	-	-	-		
1 to 5 years	27 (38.57)	7 (21.21)	0.17	0.02–1.24	0.081	-		
5 to 10 years	24 (34.29)	14 (42.42)	0.39	0.06–2.62	0.332	-		
Above 10 years	17 (24.29)	9 (27.27)	0.35	0.05–2.51	0.298	-		
**Working days per week**								
Not more than 4 days	10 (14.29)	3 (9.09)	1	-	-	1	-	-
5 days	10 (14.29)	4 (12.12)	1.33	0.24–7.56	0.745	4.04	0.47–34.69	0.203
6 days	22 (31.43)	11 (33.33)	1.67	0.38–7.32	0.499	4.34	0.74–25.60	0.104
7 days	28 (40)	15 (45.45)	1.79	0.43–7.50	0.428	4.57	0.80-26.03	0.087
**Dry cough**								
No	58 (82.86)	30 (90.9)	1	-	-	-		
Yes	12 (38.71)	3 (21.43	0.43	0.1–1.87	0.262	-		
**Productive cough**								
No	59 (84.29)	27 (81.8)	1	-	-	-		
Yes	11 (34.38)	6 (40)	1.27	0.36–4.51	0.709	-		
**Wheezing**								
No	62 (88.57)	32 (96.97)	1	-	-	-		
Yes	8 (26.67)	1 (5.88)	0.17	0.02–1.51	0.113	-		
**Morning wheezing**								
No	55 (78.57)	29 (87.9)	1	-	-	-		
Yes	15 (21.43)	4 (12.12)	0.51	0.15–1.66	0.262	-		
**Breathlessness on level ground**								
No	62 (88.57)	26 (78.79)	1	-	-	1	-	-
Yes	8 (11.43)	7 (21.21)	2.09	0.69–6.35	0.195	3.08	0.88–10.83	0.079
**Breathlessness on walking up the stairs**								
No	21 (30)	7 (21.2)	1	-	-	1	-	-
Yes	49 (70)	26 (78.79)	1.59	0.59–4.24	0.352	1.20	0.41–3.51	0.733
**Shortness of breath**								
No	52 (74.29)	21 (63.64)	1	-	-	-		
Yes	18 (25.71)	12 (36.36)	1.6	0.32–7.90	0.564	-		

^**^ Mean age (standard deviation)

COPD, Chronic obstructive lung disease; n, number; OR, Odds ratio; CI, Confidence interval; TB, Tuberculosis.

Multivariate logistic regression model adjusted for sex, BMI category, smoking cigarettes, history of TB treatment, duration of exposure per each week, breathlessness at rest and on exertion.

## Discussion

This study is the first to be conducted along coastal areas among fish vendors’ work environment (Bagamoyo and Kunduchi fish markets) assessing occupational exposure in Tanzania. In these working environments all fish vendors (100%) practice open burning in frying fish for business purposes. These findings are consistent with the Tanzania demographic health survey data which presents that more than 94% of Tanzanians use biomass fuel in cooking practices mainly charcoal and wood [29]. The use of wood has also been reported in many parts of the world as a primary cooking fuel in South Asian, African and South American countries with open fires most common in Tanzania, Pakistan, Colombia and Zimbabwe [30]. These findings are similar to many studies in Africa where availability and affordability of biomass fuel have been mentioned as a major reason for using biomass fuel [31]. The constituents of biomass smoke are known to be toxic and cause irritation of the respiratory system. These constituents include Carbon monoxide, Particulate matter, Sulfur dioxide, Nitrogen dioxide, Formaldehydes, Volatile Organic Compounds, Polycyclic Aromatic Hydrocarbons, free radicals and chlorinated dioxins [32]. Multiple risk factors for COPD have also been reported in Africa. A cigarette smoker may be exposed to air pollutants or occupational exposure [20] hence in this study multiplicity of risk factors may have resulted in the findings observed as all participants were occupationally exposed to air pollutants and 32% were cigarette smokers.

For this occupation, the number of men are higher than that of women as many men engage in fish frying. This is different from findings from other cross sectional studies conducted in different parts of the world which reported biomass smoke exposure to women and mothers who are more responsible in cooking practices at homes [33–35] while in the process of fish frying in Tanzania men are more exposed to air pollution from burning of firewood as an occupational exposure. Other studies have also reported high exposure of biomass smoke to women as they are responsible for cooking at home at their early age and hence cumulative exposure over time which lead to early manifestation of the disease [32].

Almost all fish vendors reported having respiratory symptoms (cough, breathlessness, wheezing and sputum production) which interfere with their daily activities in one way or another. The reported respiratory symptoms of this study are higher than reported findings from a cross sectional study in rural areas of Tanzania [19] which reported higher percentages of all of this symptoms with 51.7% of participants had cough, 35.6% had sputum production, 32.3% had wheezing, 25% reported breathlessness, 84.6% reported walking slower than others of same age, 69.2% they had to stop for breath when walking and 46.2% had shortness of breathing when at rest. A previous study assessing the effect of household cooking smoke reported cold as the most common symptom (46%), coughing (46.6%), phlegm production (21.2%) [36] similar to respiratory symptoms reported in this study.

The reported respiratory symptoms were similar to respiratory symptoms reported elsewhere [19, 31, 36–39]. A study done in South East Asia in Brunei Darussalam reported that cooking vendors who use biomass fuel where having higher respiratory symptoms and the symptoms were thrice more for those who have work for more than 10 years [40] same as this study where working duration of more than ten years were associated with higher respiratory symptoms. This may be because the working duration and increased working days per week increases the chance of getting higher respiratory symptoms as a results of cumulative occupational exposure overtime.

Both male and females reported experiencing higher respiratory symptoms. There are also some studies on the effect of biomass smoke and respiratory symptoms among adults for which higher risk of those symptoms reported among male same as this [20, 41] and the reason was cigarette smoking. Higher risk of respiratory symptoms among the female gender associates with the double exposure as they are responsible for cooking at homes while also exposed to biomass smoke at work. Thus the cross exposure could balance the exposure risk related to gender [20]. Some symptoms were significantly related to smoking (coughing, sputum production, wheezing and breathlessness when walking uphill) same as other studies at household level which reported an association between chronic respiratory symptoms with smoking [41, 42]. It appears that the respiratory symptoms could have results from the daily exposure to biomass smoke while also other risk factors play a role. The results of this study could be explained by the fact that firewood smoke contain many pollutants which are dangerous to health such as carbon monoxide, particulate matter, formaldehydes, sulfur dioxide, nitrogen dioxide, volatile organic compounds, polycyclic aromatic hydrocarbons, free radicals and chlorinated dioxins. Exposure to wood smoke has been associated with respiratory effects including acute and chronic changes in the lung function. Respiratory symptoms like coughing and breathlessness resulted from high exposure to firewood smoke pollutants and may aggravate lung disease and reduce the strength of the immune system. Thus alternative energy use can contribute to reduction of respiratory symptoms and COPD development. However only longitudinal studies over several years can fully account for the relationship between biomass smoke and self-reported respiratory symptoms among fish vendors.

The prevalence of current smokers and ex-smokers were higher in this study, however a cross sectional study assessing COPD in rural areas of Tanzania at household settings reported the prevalence of ex-smokers to be 25.2% higher than the one found in this study while the prevalence of current smokers were 5.4% lower than the one found in this study [19]. The previous study at household setting reported the prevalence of those who had previous history of TB to be 3.63% [19] which is higher than the one reported by this study.

No any participant had a previous spirometry test or any diagnosis of lung function same as the previous study at household level [19]. A study conducted in informal urban environment report the prevalence of COPD to be 8.13% [20] Although this is a different setting, but still the prevalence of lung obstruction in the current study based on the FEV1/FVC less than the LLN [43] is much higher as in every ten fish vendors three individual have lung obstruction. A study conducted in Malawi on use of biomass fuel among household participants found 40% of participants with abnormal spirometry but mainly restrictive lung diseases [37, 42] which is different from this study as the higher proportion of abnormal spirometry was found among participants with obstructive lung disease. The same study in Malawi found the prevalence of lung obstruction to be 8.7% (95% CI, 7.0–10.7) [42] which is lower than the findings of this study. The higher prevalence of lung obstruction among fish vendors may be because of daily exposure to firewood smoke, which may have a major public health implication in occupational settings and the community at large. Majority of participants with lung obstruction had mild to moderate lung obstruction. From these findings it shows that fish vendors are affected by firewood smoke. And since lung obstruction is progressive disease, if no immediate intervention is put in place the prevalence of respiratory symptoms will increase year to year.

The reason for observed higher prevalence may be due to occupational exposure which results from day to day exposure for 8 to 10 h. The lower numbers of females who participate in this study make generalization of findings difficult. Avoiding sex bias by including a similar number of both sex was difficult as females who participated in this occupation in Tanzania are smaller in number compared to men. The prevalence of lung obstruction in this working population suggests a hidden health problem and may be a potential for major health consequences in the future if immediate intervention is not taken into account. Major priority has to be prevention of exposure by promotion of self-awareness among fish vendors on the harmful effects of firewood smoke and among health-care workers and policy makers [44].

In logistic regression analysis age, gender, working duration, being underweight and having a previous history of tuberculosis were associated with COPD although the association were non-significant. This shows that there are some other factors responsible for lung obstruction including exposure to air pollutants of biomass smoke. As COPD was found to be associated with age, this may be due to cumulative risk over time both biologically and epidemiologically plausible thus higher risk of lung obstruction as the person ages [20, 45] but in the current study the association was non-significant. The association of lung obstruction with female gender may be explained by social cultural reasons as females are more exposed at early age in homes than males [34, 35, 46] and hence double exposure. Interestingly, participants who were obese seems to have reduced odds of COPD which is different from other studies which reported an association between obesity and COPD [47, 48].

In this study population, COPD is higher compared to previous reports in Africa as 3–4 participants out of 10 have COPD. Air pollution is a major determinant of COPD though other risk factors such as age, gender, smoking history, having previous TB history and working duration play a role. For all air pollutants measured, the average pollutant concentration before and after fish frying was below the recommended standards. Environmental air pollutants at the fish markets were higher than the WHO recommendations for both particulate matter and carbon monoxide exposure during fish frying. A study conducted in Malawi assessing air pollution at household level found the day to day air pollution exposure was approximately three times the WHO upper safety limits [42] same as the results of this study. Improving the working environment and use of clean energy remains an important strategy in controlling COPD in this population.

Findings from this study indicate that biomass use is still common in occupational settings including fish frying working environment. Health risks associated with this have been documented in many studies [13, 18, 21, 33, 45, 49] hence there is a need for immediate action to protect this workforce.

This study reports an occupational exposure to firewood smoke and respiratory symptoms among fish vendors. These findings are concurrent with other studies in Africa [19, 42, 45] which reported exposure to biomass smoke and higher respiratory symptoms among household members, among food venders and in the community. The findings of this study may be due to daily occupational exposure to firewood smoke.

Given that there is much evidence on the health effect of air pollution and the extent of public health impact of this environmental risk factor, immediate intervention to reduce the exposure and improving air quality are needed to protect public health, which require both multidisciplinary and multi-sectoral approaches.

The strength of the study is that it is the first study performed among fish vendors in Tanzania where spirometry and environmental measurements were conducted. This was a descriptive cross sectional study that described the fish vendor’s work environment, and its associated occupational exposure and the effects on the respiratory system. The study opens up for the follow up or cohort studies to be conducted in this area.

The study has some limitations, as this occupation involves more men than women, hence our results are not appropriate for some cultures where more women are involved in fish frying. As the study was only descriptive we did not measure the causal association between air pollutants and lung function values, hence a bigger study with a different design will be useful. The results of the study might also be affected by small sample size as this study uses fish vendors from Bagamoyo and Kunduchi fish markets, with only 103 participants, hence may not represent all fish vendors in the country who are exposed to biomass smoke. However, most fish vendors in the country have a similar working environment and similar exposure. The findings and reported measure of association could be limited due to the selection of the sample that shared a similar exposure from the general population.

There is also a need to detect if those with lung obstruction have a risk of progressive disease and hence there is a need for longitudinal study to observe disease progression and understand any other risk of progressive disease.

Therefore, based on the nature of the fish vendor’s working environment, it is reasonable to believe that respiratory symptoms were the result of daily exposure to air pollutants.

## Conclusions

The study found that air pollutants levels at fish vendors’ work environment during performing their activities of frying fish using firewood are higher. Further investigation is justified because the determined levels are so high and are hazardous to the health of both fish vendors and their customers. Interventions should reduce/eliminate exposure levels. This calls for the need for urgent effective approaches to reduce occupational exposure and control morbidity and mortality rate caused by firewood smoke exposure at work. There is a need for multi-sectoral and multi-stakeholder collaboration to address the problem of air pollution and NCDs and protect public health. This may involve different public and private sectors to undertake research and interventions. There is a need for enforcement and implementation of Air quality standards regulation and development of Tanzania Air quality policy. Although more evidence to validate the study is recommended, in view of this study there is a need to take measures against air pollution.

## Data Availability

The datasets used and/or analyzed during the current study are available from the corresponding author upon signing a data transfer agreement between the two parties and approval by the National Human Research and Ethics Committee of the National Institute for Medical Research.

## Abbreviations

ATS/ERS: American Thoracic Society/European Respiratory Society
BMI: Body Mass Index
COPD: Chronic Obstructive Pulmonary Disease
FEV1: Forced Expiratory Volume in one second
FVC: Forced Vital Capacity
IHI-IRB: Ifakara Health Institute- Institutional Review Board
NCDs: Non-communicable diseases
NIMR: National Institute for Medical Research
PM: Particulate Matter
WHO: World Health Organization
